# Pleiotropic function of *Dlx5/6* in the development of mammalian vocal and auditory organs

**DOI:** 10.1371/journal.pone.0337426

**Published:** 2025-12-02

**Authors:** Frida Sánchez-Garrido, Victoria Bouzerand, Markéta Kaiser, Chloé Chaumeton, Anastasia Fontaine, Tomáš Zikmund, Jozef Kaiser, Églantine Heude

**Affiliations:** 1 Laboratoire Physiologie Moléculaire et Adaptation (PhyMA), CNRS UMR 7221, Département Adaptations du Vivant, Muséum national d’Histoire naturelle, Paris, France; 2 Central European Institute of Technology, Brno University of Technology, Brno, Czech Republic; 3 Institut de Biologie Paris Seine, Sorbonne Université, CNRS, Inserm, UAR 3631, I2PS, Paris, France; 4 Institut de Génomique Fonctionnelle de Lyon, CNRS UMR 5242, École Normale Supérieure de Lyon, Université Claude Bernard Lyon 1 (UCBL), Lyon, France; University of Colorado Boulder, UNITED STATES OF AMERICA

## Abstract

Acoustic communication, a cornerstone of social interactions in mammals, relies on both vocal (effector) and auditory (receptor) organs, which display remarkable morphological diversity across species. The molecular mechanisms supporting the coordinated diversification of effector and receptor systems along with the evolution of species-specific acoustic communication are still poorly understood. A plausible hypothesis is that common genetic pathways orchestrate the parallel morphogenesis of vocal and auditory structures. Here, we addressed this question by generating mutant mice with targeted inactivation of *Dlx5/6* genes in the *Sox10* lineage, which includes neural crest and otic placode derivatives that contribute to the formation of vocal tract and ear components. We show that *Dlx5/6* inactivation led to simultaneous patterning defects in the outer, middle and inner ear and of the jaw, pharynx and larynx musculoskeletal systems. We further show that *Dlx5/6* modulate the BMP signalling pathway in both pharyngeal arches and otic vesicle, revealing a common *Dlx5/6-BMP* axis acting concurrently within the *Sox10* derivatives. These findings highlight a pleiotropic role of *Dlx5/6* in vocal and auditory morphogenesis, thereby suggesting their contribution in the co-adaptation of effector and receptor organs and in the diversification of acoustic communication in mammals.

## Introduction

Acoustic communication varies significantly among land vertebrate (tetrapod) species, ranging from ultrasonic signals in mice and bats to speech in humans. It invariably involves an effector organ, the vocal tract which produces sounds, and a receptor organ, the auditory system, that receives sounds [1–5]. Acoustic communication is critical to share information and for social interactions, such as sexual selection, maternal care or species recognition. The requirement for complementarity between sound emission and reception implies coordinated evolutionary and developmental processes for organs supporting these functions.

The vocalization process engages the coordination of a set of cranio-cervical organs including the jaws, the tongue, the pharynx and the larynx [4]. In parallel, sounds are treated through the outer (pinna), middle (ossicles) and inner (cochlea) components of the ear. In the mammalian embryo, the vocal tract originates from the pharyngeal arches (PAs) [6–10]. During early embryogenesis, these transient structures are colonized by multiple cell populations, including the cephalic neural crest cells (CNCC), the cardiopharyngeal mesoderm and mesodermal cells derived from anterior somites. CNCC give rise to the jaws, the hyoid bone and the thyroid cartilage of the larynx, and to associated connective tissues and tendons [7,10–12]. Besides that, the auditory system derives from the otic vesicle and part of the PAs. While the inner ear derives from the ectodermal otic placode, the outer ear, the ossicles of the middle ear (stapes, incus, malleus) and the tympanic ring derive from PA1–2 CNCC [13,14].

In pharyngeal arches, CNCC patterning is regulated by the *Dlx* gene family, homologous to the drosophila *distal-less* (*dll*) gene, coding for homeodomain transcription factors. In mammals, the *Dlx* family comprises three tandems of genes (*Dlx1/2, Dlx3/4* and *Dlx5/6),* with genes from the same pair showing similar expression profile and redundant functions [15–17]. During embryonic development, *Dlx5* and *Dlx6* are sequentially expressed at the lateral plate border of the neural tube, within PAs in migrating and post-migrating CNCC, in the apical ectodermal ridge of the limb bud, and later during skeletal differentiation [15,17–19]. Studies have demonstrated that *Dlx5/6* expression is required to specify mandibular identity as their constitutive inactivation leads to a transformation of the lower jaw into an upper jaw [15,19,20]. Constitutive and conditional inactivation of *Dlx5/6* in CNCC also induce defects of tongue musculature and an absence of masticatory muscles of mesodermal origin [9,15]. *Dlx5/6* genes thus play an instructive role in coordinating the formation of the jaw musculoskeletal system. *Dlx5/6*^*-/-*^ mice also present neural tube defects leading to exencephaly and to severe cranial malformations [15,20,21]. In addition, malformations of the vestibular inner ear, the tympanic ring, the middle ear ossicles, the pinna, and of the hyoid and thyroid cartilages have been occasionally described in *Dlx5/6* mutants, but the overall cranio-cervical defects have never been fully addressed [9,15,18,19,22,23].

In 1910, Ludwig Plate introduced the concept of “pleiotropy”, which refers to the influence of a single genetic locus on multiple phenotypic traits [24]. Since then, pleiotropy has been a central concept in the fields of medical genetics, evolution and developmental biology [25,26]. Theoreticians have assumed pleiotropy despite only limited empirical data on the molecular basis and relationships among multiple effects of a mutation. Given the key role of *Dlx5/6* genes in the specification of lower jaw identity, of some components of the vocal tract and of the auditory apparatus, here we investigated the potential pleiotropic role of the genes in the formation of the vocal and auditory apparatus at the basis of acoustic communication in mammals.

To do so, we generated conditional mutant mice with targeted inactivation of *Dlx5/6* genes in post migratory CNCC and in the otic vesicle using the *Sox10-Cre* driver [27,28]. Using histological approaches and X-ray computed microtomography (micro-CT), we show that *Dlx5/6* conditional inactivation induces defects of the musculoskeletal system of all organs composing the vocal tract, and of the external, middle and inner ear. By investigating morphogenetic events at the origin of malformations, our results reveal that *Dlx5/6* inactivation affects the early patterning of the otic vesicle and of CNCC derivatives within the PAs, without affecting their connective tissue fate. We also show that Dlx5/6 transcription factors regulate the expression of actors of the BMP signalling pathway in both PAs and otic vesicles. Altogether, our data unveil the pleiotropic role of *Dlx5/6* in the formation of all components of the acoustic vocal and auditory complex. We propose that *Dlx5/6* may have supported the co-adaptation of the effector and receptor organs during acoustic communication diversification in mammals.

## Materials and methods

### Animals

Procedures involving animals were conducted in accordance with European Community (Council Directive 86/609) and French Agriculture Ministry directives (Council Directive 87–848). The project was approved by the “Cuvier” ethical committee of the French National Museum of National History (MNHN) and validated by the French Ministry of Agriculture (approval APAFIS#26087-2020061614327140).

All animals were housed in light, temperature (21°C) and humidity (50%−60%) controlled conditions. Food and water were available *ad libitum*. Mice were back-crossed and maintained with a B6D2F1/J background. All genotyping was made by polymerase chain reaction (PCR) on ear biopsies.

Males carrying *loxP* sequences flanking the exon 2-containing homeobox domain of *Dlx5* and *Dlx6* genes (*Dlx5/6*^*flox/flox*^) [29] were crossed with *Sox10*^*Cre/+*^ females [28] to inactivate *Dlx5/6* (*Sox10*^*Cre/+*^: *Dlx5/6*^*flox/flox*^, *Dlx5/6* cKO). For the genetic lineage tracing, *Sox10*^*Cre/+*^: *Dlx5/6*^*flox/+*^ females were crossed with males carrying *Dlx5/6*^*flox/flox*^ and the *Rosa*^*lox-stop-lox-lacZ*^ reporter [30] (*Dlx5/6*^*flox/flox*^: *Rosa*^*lacZ/lacZ*^). For embryo and foetus collection, females were euthanized by intraperitoneal injection of a lethal dose of pentobarbital, in accordance with ethical guidelines to minimize suffering. For all experiments, the controls of mutant specimens were from the same litter. We included as control genotypes the specimens heterozygous for the Cre and/or flox or homozygous for the flox only, which we had previously validated as showing a normal phenotype compared to non-genetically modified ‘wild-type’ specimens. For *Dlx5* expression analysis, males carrying the lacZ reporter (*Dlx5*^*lacZ/+*^) [18] were crossed with B6D2F1/J females.

Samples at E10.5, E11.5 and E17.5 stages were fixed in 4% paraformaldehyde (PFA) diluted in PBS overnight at 4°C and washed in PBS. Foetuses at stage E14.5 and E16.5 were fixed for 3 hours in 4% PFA diluted in PBS and 0.5% Triton (PBSTr) at 4°C and washed twice in Tween 0.1% diluted in PBS (PBST) and then again overnight at 4°C. For all samples, noon of the day of the vaginal plug was considered as E0.5.

### X-Gal and Immunofluorescence staining

Whole-mount samples at E9.5, E10.5, E11.5 and E14.5 were collected and fixed in PFA 4% for 3 hours, washed and treated with X-gal to reveal β-galactosidase activity as previously described [7].

For whole-mount immunofluorescence staining, embryos at E11.5 were collected, fixed and dehydrated in a graded series of methanol (MeOH). They were then permeabilized in Dent’s solution (80% MeOH – 20% DMSO) overnight at 4°C and rehydrated in graded MeOH series and washed in PBS. The non-specific antigenic sites were blocked using a blocking solution (BS, PBS/ 20% goat serum/ 3% BSA/ 0.5% Triton) for 3 hours at room temperature (RT). The embryos were incubated with primary antibody diluted in BS for 2–3 days with gentle shaking at 4°C, washed several times over day in PBST and incubated with secondary antibodies also diluted in BS for 2–3 days with gentle shaking at 4°C in the dark. They were finally washed several times over day and cleared following the Cubic protocol as previously described [31].

For immunofluorescence staining on sections, foetuses were cryopreserved in 30% sucrose PBS and embedded in OCT for 20 μm sectioning using a Leica cryostat. Cryosections were dried for 30 minutes, washed in PBS and immersed in BS for 2 hours at RT. Primary antibodies were diluted in BS and sections were incubated overnight at 4°C. The sections were then washed and incubated with secondary antibodies diluted in BS for 2 hours at RT in the dark. Finally, the sections were washed in PBS and mounted with Fluoromount (Fisher Scientific, 15586276) for analysis (see S1 Table for antibody information).

### Apoptosis analysis

Whole mount samples at E11.5 were collected, fixed, dehydrated in a graded series of MeOH and stored at −20°C. The apoptotic cells were revealed using the ApoptagⓇ Fluorescein *in situ* Apoptosis detection kit (Merck, S7110) following the kit guidelines.

### Proliferation analysis

For the analysis of cell proliferation, control and mutant specimens at E12.5 were embedded in OCT, frozen, and 12 μm cryosections were prepared using a Leica cryostat. Immunostainings were then performed using anti-Sox9, anti-Desmin, and anti-PHH3 antibodies to identify chondrogenic, myogenic, and proliferating cells respectively. Confocal images were acquired using a Zeiss microscope on three consecutive sections from each region of interest. Manual counting of the total number of PHH3– and DAPI-positive cells was performed on Fiji software (ImageJ distribution, open-source) on each consecutive section. The PHH3/DAPI ratio was then determined to compare the mean number of proliferative cells in each region of interest in control and mutant conditions.

### Immunofluorescence acquisitions and analysis

Whole mount samples for lightsheet fluorescence imaging were prepared using CUBIC L-R(N) clearing protocol [31], after fixation and immunofluorescence staining. For light sheet fluorescence imaging, samples were included in agarose low melting temperature 2% (A9414, Sigma-Aldrich). We first incubated samples in 10 mL of 50% CUBIC-L reagent composed of 10% (w/w) N-butyldiethanolamine (471240, Sigma-Aldrich) and 10% (w/w) triton X-100 (Sigma-Aldrich) diluted in MilliQ water for 2 days at RT, for 2 days in 10 mL of 100% CUBIC-L at RT, and washed 3 times in PBS. They were then incubated in 10 mL of 50% CUBIC-R(N) reagent composed of 45% (w/w) Antipyrine (A5882, Sigma-Aldrich), 30% (w/w) Nicotinamide (A15970, Thermo Fisher) an 0,5% (w/w) N- butyldiethanolamine (471240, Sigma-Aldrich) diluted in MilliQ water for 2 days at RT and for 2 days in 10 mL of 100% CUBIC-L at RT. For lightsheet imaging, the samples were incubated in a fresh CUBIC-R(N) reagent in the tank and incubated for a few minutes for refractive index homogenization.

Epifluorescence imaging of embryos and foetuses *in toto* was performed with a Zeiss Macro-Apotome AxioZoom V16. Confocal imaging was made using a Zeiss LSM 980 with AiryScan 2 detector. Lightsheet imaging was performed with the Alpha3 system (PhaseView, France), equipped with a 10 × XL Plan N (XLPLN10XSVMP, Olympus, Japan) clearing objective with refractive index adaptive collar (RI 1.33–1.52), a sCMOs Orca Flash4 camera (Hamamatsu, Japan). 2.20 and the acquisition software QtSPIM. For the 561 nm excitation laser, the ET600/50m emission filter was used and for the 638 nm excitation laser, the emission filter ET670/50m (S2 Table).

### Image reconstruction and analysis

Confocal and macroscope images were reconstructed and analyzed using Fiji ImageJ (Java version 1.8.0) and ZEN Blue 3.5 (Zeiss). Airyscan and lightsheet images were converted in arivis SIS Converter 3.1.1 and analyzed with arivis Vision4D 3.0.1 software (arivis AG, Germany).

### X-ray computed microtomography (micro-CT) acquisitions and analysis

E17.5 foetuses were fixed overnight in PFA 4% and washed in several baths of PBST over day. They were dehydrated in upgrading EtOH and MeOH series and soaked in phosphotungstic acid (PTA) 1.5% diluted in MeOH 90% for 4 days. The PTA solution was changed every day with a fresh solution to ensure optimal penetration of the contrast agent. The micro-CT scanning was done by X-ray micro-CT system GE Phoenix v|tome|x L 240 (Waygate Technologies/ Baker Hughes Digital Solutions GmbH, Wunstorf) equipped with a 180 kV/15 W maximum power nanofocus X-ray tube and a high-contrast flat panel dynamic detector 41|100 with 4000 × 4000 pixels and a pixel size of 100 × 100 μm. The exposure time was 800 ms in 2000 positions over 360°. Three projections were captured in each position and an average of the signal was used to improve the signal-to-noise ratio. The microCT scan was carried out at 600 kV acceleration voltage and with 200 μA X-ray tube current. The beam was filtered by a 0.2 mm-thick aluminium filter to avoid beam hardening artefacts. The isotropic voxel size of obtained volumes was 9 μm for all four samples: 2 control and 2 *Dlx5/6* cKO. The tomographic reconstruction was performed using GE phoenix datos|x 2.0 software (Waygate Technologies/ Baker Hughes Digital Solutions GmbH, Wunstorf, Germany). Reconstructed slices were imported to software Avizo 7.1 (Thermo Fisher Scientific, Waltham, MA, USA) for semi-automatic segmentation as previously described in our previous work [32]. The muscles and other anatomical structures of interest were outlined by the operator in every 3rd to 5th slice depending on the complexity of the structure and the rest was calculated by linear interpolation between manually outlined slices. The segmented structures were then transferred to polygonal mesh (STL format) and imported to VG Studio MAX 3.5 (Volume Graphics GmbH, Heidelberg, Germany) for further visualization and analysis.

### Preparation of cDNA and RNA probes from embryos

RNA extractions were done using PureLink RNA Mini Kit with Trizol following the kit guidelines (ThermoFisher, 12183018A) and turned into cDNA libraries following the SuperScript III First-Strand Synthesis SuperMix for qRT-PCR (ThermoFisher, 11752-050) protocol. RNA probes were designed on primer BLAST (https://www.ncbi.nlm.nih.gov/tools/primer‐blast/) and synthesised by PCR, following the guidelines of Gel Extraction Kit (Qiagen, 28704), PCR Purification Kit (Qiagen, 28104) and using T7 to generate RNA probes for *in situ* hybridization.

### *In situ* hybridization

Embryos at E10.5 were collected, fixed, dehydrated in a graded series of MeOH and stored at −20°C. They were rehydrated progressively in PBST, permeabilized by Proteinase K (10 µg/ml in PBST) and post fixed in PFA 4%. Samples were equilibrated in a solution of 50% Hybridization solution (Hb) – 50% PBST 10 minutes at room temperature under agitation and in 100% Hb for an hour at 70°C. Embryos were then incubated in Hb with 1ng/µL RNA probe overnight at 70°C.

After 4 successive washes of 30 minutes with Wash Buffer (WB) at 70°C, they were equilibrated in a graded series of TBST at room temperature. Embryos were blocked with Blocking Buffer (BB) and incubated overnight at 4°C in Antibody Solution (AbS) containing anti-DIG antibodies coupled with alkaline phosphatase (Merk, 11093274910). Samples were washed in TBST several times followed by 3 NTMT washes and revealed with BM-Purple (Merk, 11442074001) until they reached proper staining contrasts. Composition of solutions are presented in (S3 Table).

## Results

### *Dlx5/6* inactivation affects the entire vocal tract and auditory systems

To induce targeted inactivation of *Dlx5/6* in CNCC and otic vesicle (*Dlx5/6* cKO), we crossed *Sox10*^*Cre/+*^ deleter mice with the previously-described *Dlx5/6*^*flox/flox*^ mouse line [9,29], in which both the homeodomain-encoding region (exon 2) of *Dlx5* and *Dlx6* are flanked by *loxP* sequences. *Sox10* and *Dlx5/6* genes are both expressed in post migrating CNCC populations, in the otic placodes and their derivatives (S1 Fig) [15,18,27,28].

**Fig 1:**
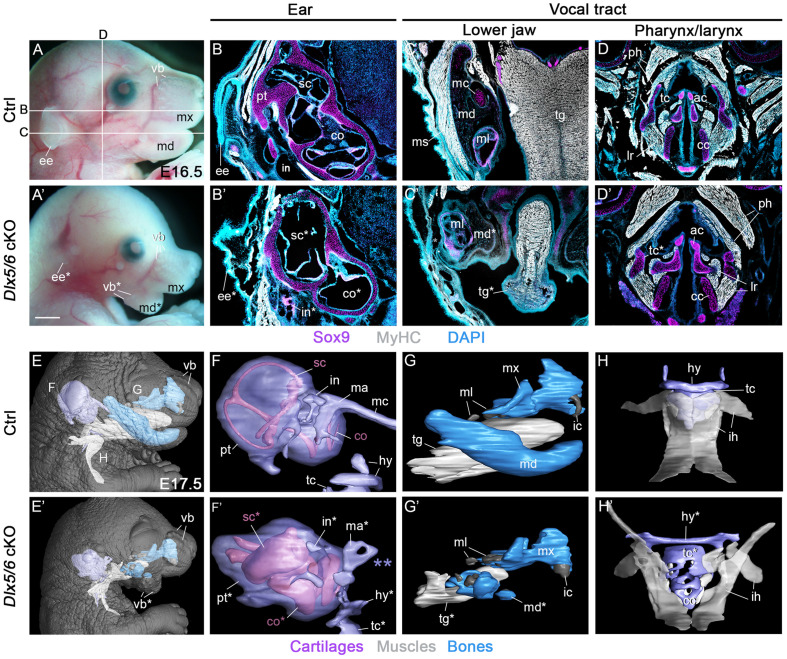
*Dlx5/6* genes are required for the proper formation of organs composing both the vocal and auditory systems. (A–A’) Macroscopic view of control and *Dlx5/6* cKO mutant phenotypes at E16.5. In mutants, the lower jaw is defective with ectopic vibrissae (vb*) and the external ear (pinna) shows severe hypoplasia. (B–D’) Immunofluorescence stainings for the cartilage marker Sox9 and muscle marker MyHC on coronal and frontal sections at the levels indicated in A. (B–B’) The *Dlx5/6* mutant presents malformations of external, middle and inner ear components including reduced pinna, defective incus and semicircular canals, and enlarged cochlear duct. (C–D’) The *Dlx5/6* mutant presents a transformed lower jaw characterized by an absence of Meckel cartilage and of associated masticatory musculature. The tongue is severely reduced with disorganized myofibers. The thyroid cartilage also seems reduced compared to control (n = 6 each condition, control genotype: *Dlx5/6*^*flox/flox*^). (E–H’) 3D reconstructions of the different structures composing the vocal tract and auditory systems in control and *Dlx5/6* mutant conditions at E17.5 (n = 2 each condition, control genotype: *Dlx5/6*^*flox/flox*^ and *Dlx5/6*^*flox/+*^). (F–F’) The Meckel cartilage is missing (purple double asterisk in F’), the middle ear ossicles (incus and malleus) are defective and the vestibular and cochlear membranous labyrinths are swollen in mutants compared to controls. (G–H’) The lower jaw transformation in mutants is associated with a hypoplastic tongue and mispatterned extrinsic laryngeal (infrahyoid) muscles. The thyroid and hyoid cartilages also show defects of formation in the mutant context. All structures presenting a malformation in the *Dlx5/6* mutant context are indicated with an asterisk. Absent or reduced structures are noted with a double asterisk. See also S1 and S2 Appendices for interactive PDFs of 3D reconstructions. Abbreviations: ac, arytenoid cartilage; cc, cricoid cartilage; co, cochlea; ee, external ear; hy, hyoid cartilage; ic, incisor; in, incus; lr, laryngeal muscles; ma, malleus; mc, Meckel cartilage; md, mandible; ml, molar; ms, masseter muscle; mx, maxilla; pt, petrous part of the temporal bone; ph, pharyngeal muscles; sc, semicircular canals; tc, thyroid cartilage; tg, tongue; vb, vibrissae. Scale bar in A’ for A-A’ 1000 µm, for B-D’ 200 µm.

The macroscopic phenotypic analysis before birth revealed that *Dlx5/6* cKO foetuses presented craniofacial malformations similar to those observed in previous *Dlx5/6* inactivated mouse models [9,15,18,19], including a transformed lower jaw exhibiting ectopic vibrissae and hypoplastic external ear (Fig 1A–A’).

First, we performed immunofluorescence staining for MyHC and Sox9 on sections to investigate respectively the muscular and skeletal phenotypes of control and mutant foetuses at E16.5. Regarding the auditory system, Sox9 immunostainings revealed an hypoplasia of the middle ear ossicles of CNCC origin [13] (Fig 1B–B’). The inner ear showed defects of the membranous and bony labyrinths of the semicircular canals and cochlea (Fig 1B–B’). At the level of the vocal tract, we observed that the transformed lower jaw was associated with tongue hypoplasia and masticatory masseter muscle aplasia (Fig 1C–C’), as previously described after *Dlx5/6* inactivation [9,15]. The hyoid and the laryngeal thyroid cartilages derived from CNCC [7,12] seemed also impacted but the extent of malformation was difficult to access on sections.

To push forward the phenotypic analysis, we performed micro-CT scanning of mutant and control foetuses at E17.5 to access the complex layout of the craniocervical structures and to reconstruct in 3D the different components of the vocal tract and auditory system (Fig 1E–H’, S1 and S2 Appendices). This approach clearly revealed the extent of inner ear malformations including swollen membranous labyrinths with indiscernible semicircular canals and wider cochlear duct (Fig 1F–F’). Moreover, we observed the presence of two small mispatterned skeletal structures at the site of the incus and malleus ossicles associated with an absence of Meckel cartilage in mutants (Fig 1F–F’). The 3D reconstruction of the jaw musculoskeletal elements highlighted the severe malformations of the lower jaw (mandible) and of the tongue musculature (Fig 1G–G’). The hyoid and thyroid cartilages were severely affected and extrinsic laryngeal (infrahyoid) muscles showed patterning defects (Fig 1H–H’). Pharyngeal and intrinsic laryngeal muscles presented morphology differences compared to controls adapted to the defective hyoid and thyroid cartilages to which they connect. In contrast, the cricoid and arytenoid cartilages of mesodermal origin [7,12] appeared unaffected by *Dlx5/6* mutation (Fig 1D–D’, S1 and S2 Appendices).

### *Dlx5/6* genes regulate early CNCC and otic vesicle patterning

**Fig 2:**
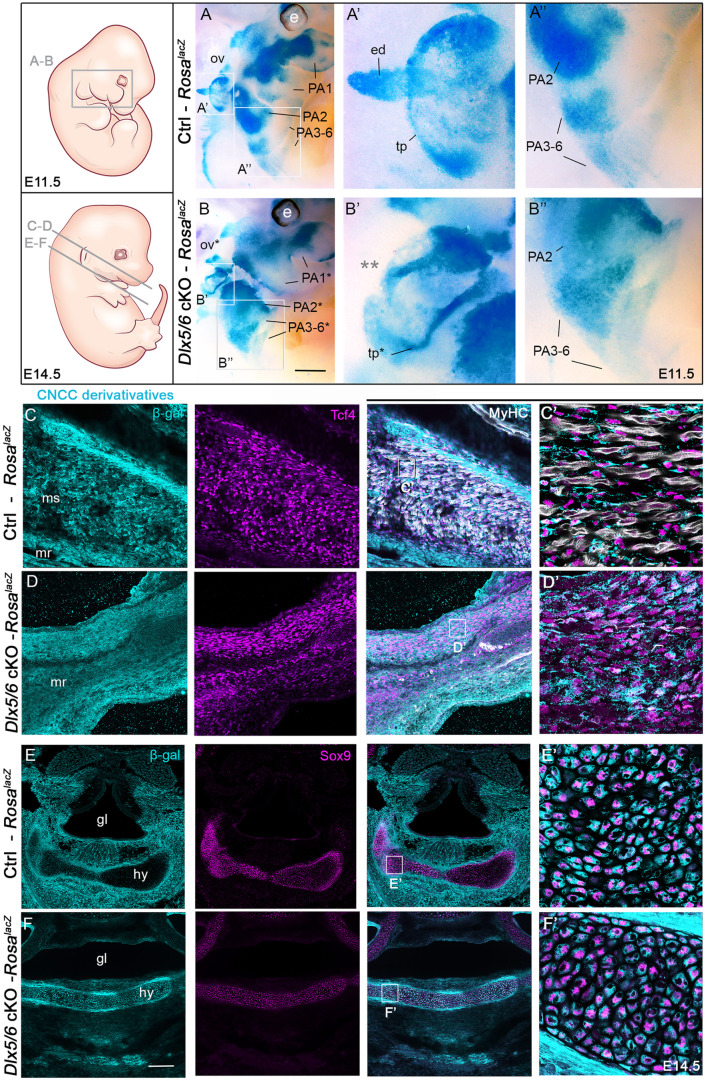
*Dlx5/6* regulate the patterning of CNCC in the PAs and of the otic vesicle without affecting their connective tissue fate. **(A-B)** *Sox10* genetic lineage tracing of CNCC and otic placode derivatives using the Rosa-lacZ reporter in control and *Dlx5/6* mutant embryos at E11.5. In mutants, the otic vesicle shows a lack of endolymphatic sac (grey double asterisk in B’) and defect of the tympanic ring compared to controls (A’–B’). β-gal-positive CNCC compose the mesenchymal population of the PAs in controls that show aberrant patterning in mutants (A”–B”). (C–F’) Immunofluorescence stainings on sections for β-galactosidase labelling CCNC derivatives, and for Tcf4, MyHC and Sox9 that mark connective tissue fibroblasts, muscles and cartilages respectively in control and mutant foetuses at E14.5. Tcf4-positive fibroblasts of CNCC origin are detected between masticatory myofibers associated with the lower jaw in controls (C–C’). β-gal/Tcf4-positive fibroblasts are maintained in the masticatory region of mutants despite the absence of differentiated musculature (D–D’). At the level of the glottis and hyoid cartilage, we observe differences in the distribution of CNCC derivatives that still keep their cartilaginous identity (E–F’). All structures presenting a malformation are marked with an asterisk. Absent or reduced structures are noted with a double asterisk (n = 3 each condition, control genotype: *Sox10*^*Cre/+*^; *Dlx5/6*^*flox/+*^; *Rosa*^*lacZ/+*^). Abbreviations: ed, endolymphatic sac; gl, glottis; hy, hyoid cartilage; mr, masticatory region; ms, masseter muscle; ov, otic vesicle; PA, pharyngeal arch; tp, tympanic ring. Scale bar in B for A–B 400 µm, for A’–B’ 150 µm, for A”-B” 200 µm, in F for C–F 200 µm, for C’–F’ 20 µm.

We then used genetic lineage tracing approaches to analyze the morphogenetic events at the basis of the musculoskeletal malformations in *Dlx5/6* mutants. We first examined the fate of CNCC by *Sox10* genetic lineage tracing using the *Rosa*^*lox-stop-lox-lacZ*^ (*Rosa*^*lacZ/+*^) [30] reporter analysis at E11.5 in control and mutant conditions (Fig 2A, B). β-galactosidase (β-gal) positive cells corresponding to placode derivatives, were detected in the tympanic ring and the endolymphatic sac of the otic vesicle in controls (Fig 2A–A’). We also observed β-gal-positive cells corresponding to CNCC derivatives within the mesenchyme of the maxillary and mandibular parts of the PA1, the hyoid arch (PA2) and more posterior arches (PA3–6) (Fig 2A”), which later give rise to the skeletal components of the jaws, the ossicles and the hyoid and thyroid cartilages. In *Dlx5/6* cKO mutants, both compartments showed organization defects of β-gal-positive cells. In the developing ear, the endolymphatic sac was missing and the tympanic ring was mis-patterned, whereas β-gal-positive CNCC were scattered and disorganized within the PA1–6 (Fig 2B–B”). We did not detect any obvious differences between controls and mutants in cell apoptosis within the pharyngeal arches at E11.5, nor in cell proliferation within otic and vocal components at E12.5 (S3 Fig). Remarkably, no additional defects were observed along the body axis, indicating that our targeted *Dlx5/6* mutation specifically affects vocal tract and auditory precursors.

We then followed the fate of β-gal derivatives during musculoskeletal differentiation at E14.5. We performed immunofluorescence stainings on sections for anti-β-gal and for markers of cartilaginous, fibroblastic or tendinous derivatives (Sox9, Tcf4 and Tnc respectively) of placodal and CNCC populations. In controls, CNCC-derived β-gal cells corresponded to Tcf4-positive fibroblasts forming the muscle connective tissue along the myofibers of the masticatory masseter and tongue muscles (Fig 2C–C’, S2A Fig) [7]. In mutants, the β-gal/Tcf4-positive muscle connective tissue fibroblasts are present, even in the absence of masseter musculature (Fig 2D–D’, S2B Fig). The β-gal/Tcf4-positive populations of the external ear were also preserved in the hypoplastic pinna of mutants that however showed defects in organization compared to controls (S2C, S2D Fig). The β-gal-positive CNCC-derived population showed aberrant patterning at the level of the glottis, the hyoid and thyroid cartilages but kept their cartilaginous and tendinous identity as observed by Sox9, Tnc and β-gal colocalization in the structures of both controls and mutants (Fig 2E–F’, S2E–S2H Fig).

**Fig 3:**
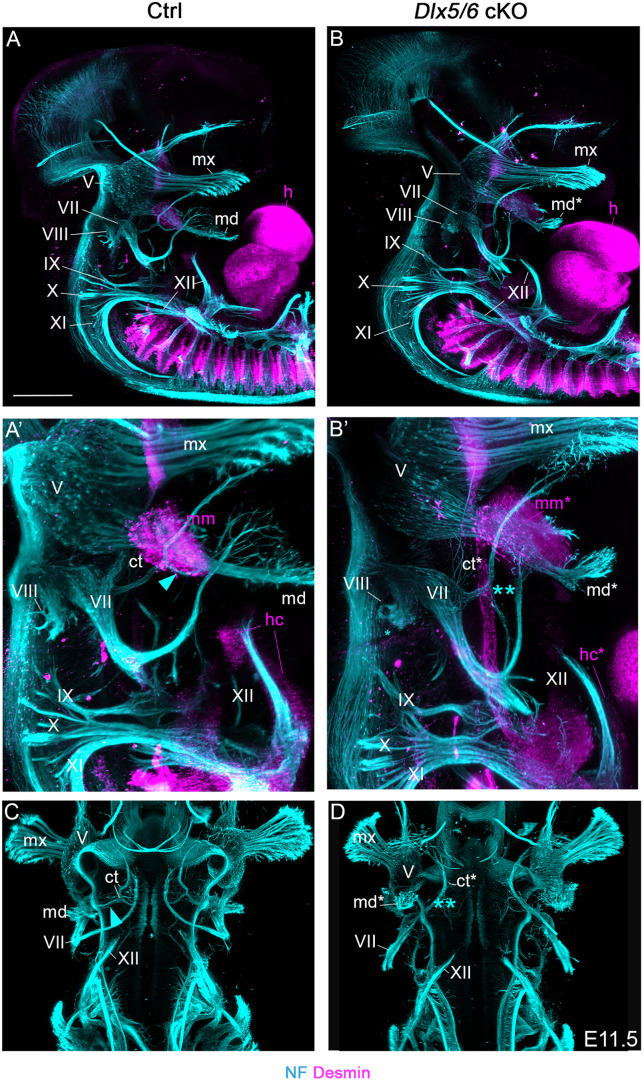
*Dlx5/6* inactivation induces innervation defects of some vocal and auditory components. **(A–D)** *In toto* immunofluorescence staining of control and mutant embryos at E11.5 for the neurofilament (NF) and Desmin that label the peripheral nervous system and muscle precursors respectively. Lateral (A–B’) and ventral (C–D) views with magnification at the level of vocal tract and auditory precursors (A’–B’). In mutants, the mandibular branch of the trigeminal nerve (V) presents characteristics of the maxillary branch with increased distal arborization to connect ectopic vibrissae. The vestibulocochlear nerve (VIII) shows defective projection toward the otic vesicle. The *chorda tympani* branch of the facial nerve (VII) (blue arrowheads in controls A’, C) misses the neuronal projection that later innervates the tongue (blue double asterisk) in mutants B’, D). Note that the masticatory muscle precursor is present at E11.5 while the hypoglossal cord giving rise to tongue muscles is already reduced in mutants. All structures presenting a mutant phenotype are noted with an asterisk. Absent or reduced structures are noted with a double asterisk (n = 3 each condition, control genotype: *Sox10*^*Cre/+*^; *Dlx5/6*^*flox/+*^). See also S1 and S2 Movies for interactive view of the neuromuscular system in control and mutant conditions. Abbreviations: ct, *chorda tympani*; h, heart; hc, hypoglossal cord; md, mandibular branch of the trigeminal ganglion; mm, masticatory muscle precursor; mx, maxillary branch of the trigeminal ganglion. Scale bar in A for A-B, C-D 300 µm, for A’-B’ 200 µm.

We then investigated whether such alterations in *Dlx5/6* expression could also affect the innervation of vocal tract and auditory components. We analyzed the neuromuscular system of control and mutant embryos by *in toto* immunofluorescence stainings at E11.5 targeting the neurofilament (NF) and Desmin proteins that mark respectively the developing peripheral nervous and muscular systems (Fig 3). In controls, the maxillary and mandibular branches of the trigeminal ganglion (cranial nerve CN V) project into the upper and lower jaw buds to innervate notably the sensory vibrissae and the masticatory muscle precursor (Fig 3A–A’). We also observed the facial nerve (CN VII) that innervates the facial muscles and its *chorda tympani* branch that later join the lingual nerve (CN V) in the mandibular arch [33]. More dorsally, the vestibulocochlear nerve (CN VIII) innervates the inner ear, and posteriorly the glossopharyngeal, the vagus and accessory nerves (CN IX, X, XI) project to innervate the tongue, throat and laryngeal components (Fig 3A–A’, S1 Movie) [34]. In *Dlx5/6* cKO, the mandibular branch of the trigeminal ganglion shows increased distal arborization characteristic of the maxillary branch to innervate the ectopic vibrissae observed on the transformed lower jaw (Figs 1A’, E’, 3B, S4 Fig). The mandibular neuronal projection toward the masticatory muscle precursor is also reduced in E12.5 mutants (S4 Fig). The vestibulocochlear nerve (CN VIII) shows defect of projection on the otic vesicle in the mutants compared to controls (Fig 3B–B’). Moreover, one branch of the *chorda tympani* that innervates the distal part of tongue later during development was missing. However, we did not detect any obvious differences in the hypoglossal nerve (CN XII) while the hypoglossal cord forming the tongue was already reduced in E11.5 mutants. In contrast, the masticatory muscle precursor was present at this stage as previously described in constitutive *Dlx5/6* mutants, the defect of masticatory muscle differentiation occurring during late embryonic stages in mutants (Fig 2, S4 Fig) [15]. We did not notice differences in the configuration of the glossopharyngeal, the vagus and accessory nerves (CN IX, X, XI) between controls and mutants (Fig 3B’–D, S2 Movie).

Altogether, our results show that *Dlx5/6* expression concurrently regulates the proper patterning and the innervation of both PA and otic placode derivatives, without altering their connective tissue fate during the development of the vocal tract and auditory components.

### Dlx5/6 modulate BMP signaling in both pharyngeal arches and otic vesicle

It was previously reported that several actors of the BMP signaling pathway are downstream targets of Dlx5 in the otic vesicle [35]. Some BMP signaling genes were also shown to be expressed during pharyngeal arch patterning and affected by *Dlx5/6* constitutive inactivation [36]. We thus wondered if Dlx5/6 transcription factors may act as regulators of the BMP signaling pathway in both vocal tract and auditory precursors.

We selected key genes, *Bmper, Msx1* and *Hand2*, and analyzed their expression by *in situ* hybridization during early development at E10.5. Bmper (BMP-binding endothelial cell precursor-derived regulator) is a secreted protein present in migrating CNCC and the mesodermal core of PAs [36–39]. It was reported that *Bmper-*inactivated mice showed malformations of the thyroid cartilage [38]. Msx1 and Hand2 are downstream components of the BMP signaling cascade, they are expressed in CNCC and are regulated by *Dlx5/6* in pharyngeal arches [36,40,41]. *Msx1* and *Hand2* deficient mice show malformations of the middle ear, the hyoid and thyroid cartilages [42,43].

**Fig 4:**
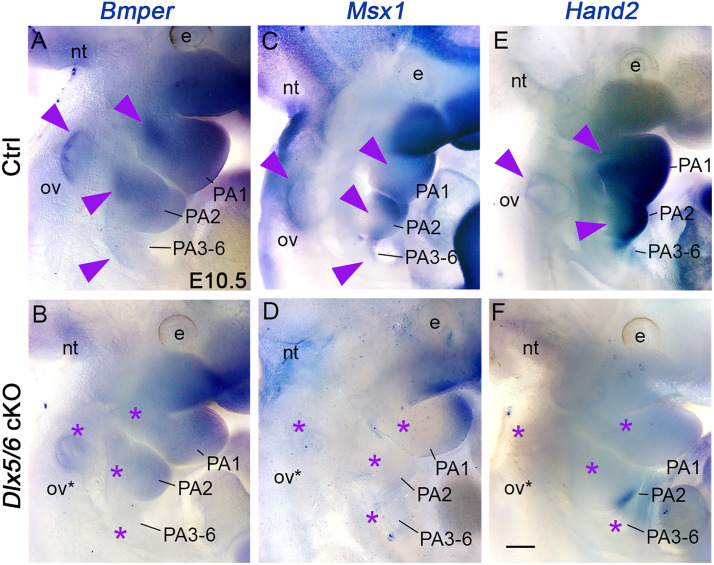
Dlx5/6 transcription factors regulate BMP signaling in both pharyngeal arches and otic vesicle. Whole mount *in situ* hybridization for *Bmper*, *Msx1* and *Hand2* in E10.5 control and mutant embryos **(A–F)**. *Bmper* expression is observed in the mesodermal core and the distal part of PA1-2 and in the tympanic ring of controls **(A)**. In mutants, *Bmper* expression is lost in the mesodermal cores and expression is reduced in the distal PAs and otic vesicle **(B)**. In controls, *Msx1* and *Hand2* are expressed in all PAs and otic vesicles (arrowheads in C, **E**). In mutants, expression is undetectable in the otic vesicle and reduced or undetectable in PAs **(D, F)**. All structures presenting undetectable or reduced gene expression in the mutant are marked with an asterisk (n = 4 each condition, control genotypes: *Dlx5/6*^*flox/+*^ and *Dlx5/6*^*flox/flox*^). Abbreviation: e, eye; nt, neural tube; ov, otic vesicle; PA, pharyngeal arch. Scale bar in F for A-F 200 µm.

All the genes analyzed were expressed in both otic vesicle and pharyngeal arches at E10.5 in control embryos (Fig 4). *Bmper* was expressed in the tympanic ring of the otic vesicle and in the mesodermal core of the PA1–2 (Fig 4A). Expression was also noticed in the distal part of PA1–2 and in PA3–6. In *Dlx5/6* mutant embryos, expression in the distal PA1–2 and otic vesicles was reduced and undetectable in the PA1–2 mesodermal cores and posterior arches (PA3–6) (Fig 4B).

In the control, *Msx1* was expressed in the distal cap of PA1–2, in posterior PAs, as well as the otic vesicle (Fig 4C). In our mutants, *Msx1* expression was lost in PA2–6 and otic vesicles, and reduced in PA1 (Fig 4D). *Hand2* was strongly expressed in all pharyngeal arches and expression was detected in the otic vesicle in controls (Fig 4E). The *Dlx5/6* mutant embryos showed a drastic loss of expression in both compartments (Fig 4F). Our analysis indicates that *Dlx5/6* genes directly regulate the BMP signaling pathway through *Bmper* early during the development of both vocal tract and auditory precursors.

## Discussion

### A common Dlx5/6-BMP axis acts in *Sox10* derivatives during auditory and vocal tract development

In this study, we show that Dlx5/6 transcription factors control the early patterning and innervation of pharyngeal arch and otic derivatives by regulating the BMP signaling pathway in both compartments.

The BMP signaling pathway is implicated in many developmental processes including cellular differentiation, growth and apoptosis [44]. It has been previously shown that *Dlx* genes and BMP signaling interact for proper craniofacial development [22,45]. In our study, we selected and analyzed actors of the BMP signaling pathway that were shown to be direct downstream targets of *Dlx5* in the otic placode [35] and that we noticed being also expressed in both PAs and otic vesicles. We observed that the gene coding for the secreted protein Bmper (*Cv2*) was expressed in distal CNCC and within the mesodermal cores of PA1–2 in controls. In our mutants, *Bmper* expression was reduced in CNCC and not detectable in the mesodermal cores in which *Dlx5/6* is not expressed, indicating indirect regulation of *Bmper* expression by *Dlx5/6* in the latter compartment. We previously demonstrated that *Dlx5/6* expression in CNCC is necessary to instruct the adjacent mesoderm for the proper differentiation of masticatory muscles [9,15], but the molecular actors involved in CNCC-mesoderm interaction remained elusive. We hypothesize that the secreted Bmper, expressed in both CNCC and mesodermal populations and regulated by Dlx5/6, may handle such a role. *Bmper*-inactivated mice show defects of laryngeal cartilages and hypoplastic skull vault, however the head muscular phenotype had not been investigated [38]. Further functional analyses in the mouse embryos are still required to test if Bmper could be a mediator between Dlx5/6 in CNCC and the adjacent mesoderm to regulate head muscle differentiation.

We have also shown that inactivation of *Dlx5/6* severely impacts the expression of *Hand2* and *Msx1* in both otic vesicles and PAs. These genes are known to act downstream of the BMP signaling and it has been proposed that *Dlx5/6* regulate their expression to direct lower jaw patterning [36,41]. Moreover, Chip-seq data have identified *Msx1* as a direct target of Dlx5 in the inner ear precursor [35]. The data indicate that *Hand2* and *Msx1* are regulated directly or indirectly by *Dlx5/6* during PA and otic vesicle development.

It has been shown that the Hedgehog (HH) signalling pathway, including its key transducer Gli3, controls larynx formation and function [12,46]. Notably, *Shh* and *Gli3* mutants show patterning defects of CNC derivatives forming the laryngeal connective tissue, correlated with changes in the acoustic structure of vocalizations [12]. A study demonstrated that Gli3 and Hand2 transcription factors act in a synergistic manner in cephalic neural crest derivatives for proper skeletal and glossal development [47]. HH signaling and the BMP pathway regulated by Dlx5/6 may work together to control laryngeal development.

**Fig 5:**
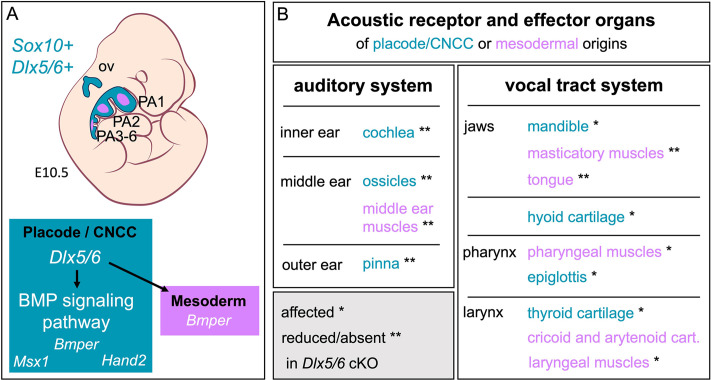
A common Dlx5/6-BMP axis acts in *Sox10* derivatives during the formation of both auditory and vocal tract systems. **(A)** Schematic views of *Sox10* and *Dlx5/6* co-expression in the otic vesicle (ov) and pharyngeal arches in E10.5 mouse embryos (in blue), and role of *Dlx5/6* in regulating BMP signaling in placode and CNCC (in blue) and in PA mesodermal core derivatives (in pink). **(B)** Summary of the phenotype resulting from *Dlx5/6* conditional mutation affecting both acoustic receptor and effector organs of placode/CNCC (in blue) or mesodermal origins (in pink).

In contrast to *Wnt1* that is an early and specific marker of neural crest derivatives, the *Sox10* lineage also includes the otic vesicle [28]. In chick, a common enhancer element controls *Sox10* expression in both the otic placode and CNCC [27], indicating that the two compartments are co-regulated by Sox10 activation during early development. Altogether, the data thus suggest that a common *Dlx5/6-BMP* genetic program acts within the *Sox10* lineage during the morphogenesis of the vocal and auditory complex (Fig 5).

### *Dlx5/6* pleiotropic role may have supported acoustic communication diversification

The *Dlx* gene family has an ancient origin in bilaterians and is present across all vertebrate clades [16]. Expression analyses in chick, xenopus and fish embryos revealed that *Dlx5/6* expression in the pharyngeal arches and otic vesicle is conserved across vertebrate species [48–52]. Our data show that *Dlx5/6* genes simultaneously regulate the formation of all structures composing the vocal tract and auditory systems (Fig 5), receptor and effector organs of acoustic communication. This fully corresponds to the concept of pleiotropy since a single locus affects distinct phenotypic traits [24,25].

Research has demonstrated that acoustic communication originated once in the land vertebrates [1,2] and that the larynx is the main site of vocal production within the vertebrate family [4]. The mammalian clade comprises diverse ecological niches, a diversity that is explained by the rapid adaptive radiation during “the age of mammals” and terrestrial changes like continental rearrangements [4,53]. Mammals show a wide variety of acoustic transmission varying from infra to ultrasounds, possibly originating from the adaptation to new environments and for predator avoidance [54]. The differences in tone and frequency can be explained by the morphological diversity of the larynx in mammals as well as by the adaptation of the receptor organ capable of hearing infra or ultrasounds [3,4]. It has been suggested that mammalian vocal characteristics and hearing sensitivity co-evolved in the forest mammals, following the sensory drive hypothesis [55]. One of the characteristics of the mammalian clade is the presence of ossicles of CNCC origin. Their morphology is dependent on environment and behavior making the auditory apparatus competent to pick up a wider variety of frequencies [54]. Beyond sound emission and reception, a study revealed that targeted inactivation of *Dlx5/6* in GABAergic neurons results in increased vocalization and socialization behaviors in adult mice [56]. We thus propose that the pleiotropic role of *Dlx5/6* genes might have been involved in the co-adaptation of vocal and auditory acoustic organs, but also in the regulation of cognitive communication capacities in mammals.

Our *Dlx5/6* mutants display malformations that also affect traits of domestication [57]. It has been proposed that the “Neural Crest Domestication Syndrome” (NCDS) results from a reduction of CNCC-derived tissues of behavioral relevance and inducing morphological changes of the jaw, ear or larynx [57–59]. In domesticated mammalian species such as cats and dogs, pets interact with humans with acoustic emissions that are not recorded in the wild. The pleiotropic role of *Dlx5/6* in development and cognition may have supported the morphological changes predicted by the NCDS hypothesis by adapting both the auditory and vocal systems and by inducing a domesticated behavior [56].

The origin of speech in humans has been approached through anatomical analyses of the brain, the face and the vocal tract and, more recently, through the comparison of genes involved in language acquisition [60,61]. It has been shown that genes associated with face and vocal tract anatomy went through particularly extensive methylation changes in modern humans after the split from Neanderthals and Denisovans [61]. It has been proposed that *DLX5* had played a role in the evolution of the human’s linguistic capacity and skull globularization, and that *DLX5/6* had contributed to human “self-domestication” [56,60]. Interestingly, the *DLX5/6* locus shows methylation motifs that changes linearly with age in mouse and human tissues including the brain [56]. The concomitant modifications of craniofacial morphology, cognition, and language abilities may have been controlled by a common genetic network including *DLX5/6* genes during human evolution.

### Incidence for understanding the etiology of human syndromic forms

In humans, the Split Hand/Split Foot malformation 1 (SHFM1, OMIM #183600) is characterized by ectrodactyly caused by pathogenic variants affecting the *DLX5/6* locus [62]. *Dlx5/6* mutant mice have been investigated to understand the etiology of the limb phenotype in SHFM1 [63]. In patients, the limb malformations can be associated to other defects including cranial malformations, hearing loss and intellectual disabilities (OMIM #220600). It has been proposed that a minimal SHFM1 chromosomal region containing *DLX5/6* enhancers would be compromised and related to associated phenotypes [62]. Moreover, the *DLX5/6* intergenic regulatory sequence includes SNPs associated with either hyper-(Williams–Beuren syndrome) or hypo- (ASDs) sociability and vocalization syndromes [56]. The pleiotropic role of *Dlx5/6* in vocal tract and otic development, and in socialization and vocalization abilities is thus relevant to better understand the hearing loss, craniofacial features and cognitive traits associated with the limb phenotype in SHFM1 patients.

## Supporting information

S1 Fig *Dlx5* and *Dlx6* expressions during mouse development. (A-F) *In situ* hybridization for *Dlx5* and *Dlx6* at E9.5, E10.5 and E11.5 in control embryos. Note that the genes show similar spatio-temporal expression profiles within the PA CNCC and otic vesicle. (G-J) Expression of the *Dlx5-lacZ* reporter recapitulates the expression profiles of *Dlx5* and *Dlx6* in E9.5, E10.5, E11.5 embryos, but showing better signal in the posterior PAs (PA3–6). In clarified E14.5 *Dlx5*^*lacZ/+*^ foetuses, β-gal expression is activated in the developing skeleton. Magnifications of the ear in (J’) and of the laryngeal regions in (J”) (n = 3 each condition). Abbreviations: e, eye; hy, hyoid cartilage; lb, limb; lr, laryngeal cartilages; mb, mandible; mx, maxilla; ov, otic vesicle; PA, pharyngeal arch; sc, semicircular canals; vt, vertebrae. Scale bar in F for A, D, G 150 µm, for B, E, H 200 µm, for C, F, I 300 µm, for J 500 µm, for J’-J” 150 µm.
(TIF)

S2 Fig Inactivation of *Dlx5/6* affects CNCC patterning but not CNCC fate. (A-H) Immunofluorescence stainings for β-gal and for Tcf4 and Tnc, markers of CNCC-derived fibroblasts and tendons at the tongue, external ear and thyroid levels in control and *Dlx5/6* mutant conditions. (A-B, E-F). In the tongue, β-gal-positive cells show defects of patterning but keep their fibroblast and tendinous identities (C-D), as they do at external ear and thyroid levels (C-D, E-H) (n = 3 each condition, control genotypes: *Dlx5/6*^*flox/+*^; *Rosa*^*lacZ/+*^). Scale bar in F for A-H 20 µm.
(TIF)

S3 Fig Apoptosis and proliferation analysis in control and mutant embryos. (A) TUNEL assay on whole-mount control and mutant embryos at E11.5. A Similar number of apoptotic cells were identified within the maxillary and mandibular prominences in control and mutant specimens (n = 3 each condition, control genotype: *Dlx5/6*^*flox/flox*^). Abbreviations: h, heart; PA1–2, pharyngeal arches 1–2. Scale bar in A 200 µm. (B) Quantitative analysis indicates no significant difference using the non-parametric Mann–Whitney test in the proportion of PHH3-positive proliferative cells between E12.5 control (n = 2, control genotype: *Sox10*^*cre/+*^; *Dlx5/6*^*flox/+*^) and mutant (n = 3) specimens across the regions of interest shown in panels as examples. Scale bar in B 20 µm. Raw data quantifications are presented in (S4 Table).
(TIF)

S4 Fig Phenotype of *Dlx5/6* mutants at late embryonic developmental stage. (A-B) *In toto* immunofluorescence staining of control and mutant embryos at the E12.5 for Sox9, MyHC and NF that label the developing cartilaginous, muscular and nervous systems respectively, at the level indicated on the scheme at the top. (A’-B’) In mutants, the chondrogenic condensations of the otic vesicle, the pharynx and the larynx appear already defective while the Meckel cartilage is undetectable compared to control (B’, white asterisk). (A”-B”) The mutant also presents a severe reduction of the masticatory muscle precursor and tongue defects, while the neck and heart musculature appear normal. (A”’-B”’). At late embryonic stage, the vestibulocochlear nerve (VIII) is slightly reduced, and the transformed mandibular branch of the trigeminal nerve shows aberrant distal arborization associated with a defect of neuronal projection towards the masticatory muscle precursor (B,” white asterisk). All structures presenting a mutant phenotype are noted with an asterisk (n = 2 each condition, control genotype: *Dlx5/6*^*flox/+*^). Abbreviations: cv, cervical vertebrae; h, heart; lb, limb; lr, larynx region; mc, Meckel cartilage precursor; md, mandibular branch of the trigeminal ganglion; mm, masticatory muscle precursor; mx, maxillary branch of the trigeminal ganglion; nm, neck muscles; ov, otic vesicle; ph, pharynx region; tg, tongue precursor. Scale bar in B”’ for A-B”’ 400 µm.
(TIF)

S1 Table List of primary and secondary antibodies used in the study. (PDF)

S2 Table Microscopes and acquisition parameters. (PDF)

S3 Table Composition of solutions used for *in situ* hybridization. (PDF)

S4 Table Raw data of PHH3 and DAPI cells quantification shown in S3 Fig. (PDF)

S1 Appendix Interactive 3D PDF of the control phenotype reconstructions of vocal tract and auditory systems to complete data shown in Fig 1E–H’.
(PDF)

S2 Appendix Interactive 3D PDF of the *Dlx5/6* mutant phenotype reconstructions of vocal tract and auditory systems to complete data shown in Fig 1E–H’. (PDF)

S1 Movie Interactive movie of the neuromuscular system of a control embryo to complete data shown in Fig 3.
(MP4)

S2 Movie Interactive movie of the neuromuscular system of a Dlx5/6 mutant embryo to complete data shown in Fig 3.
(MP4)
