# Supplementary figures and images for: Pleiotropic function of Dlx5/6 in the development of mammalian vocal and auditory organs
Source: PLoS One. 2025 Dec 2;20(12):e0337426. doi: 10.1371/journal.pone.0337426 (PMC12671821; doi:10.1371/journal.pone.0337426)

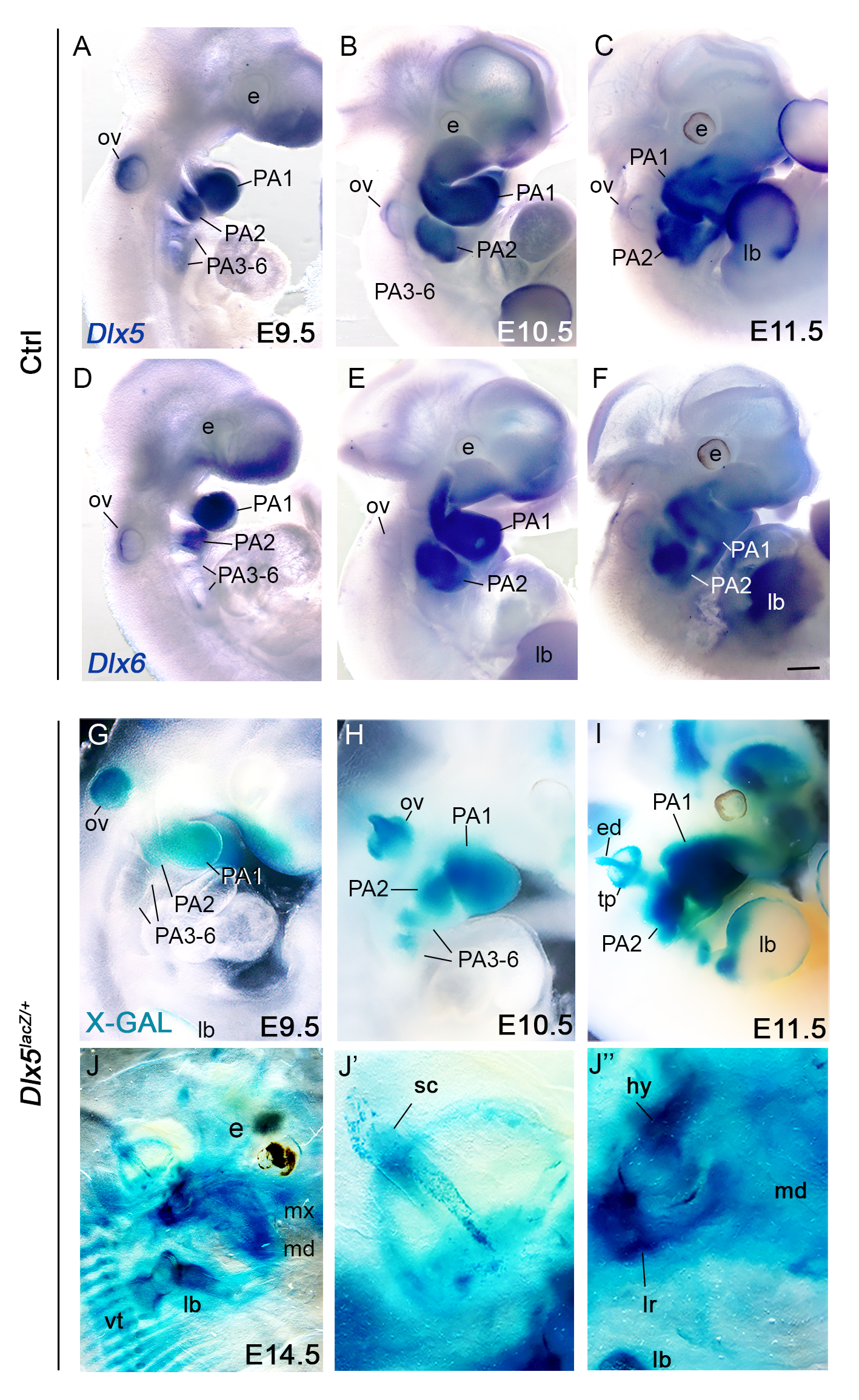

Supplement: S1 Fig — (A-F) In situ hybridization for Dlx5 and Dlx6 at E9.5, E10.5 and E11.5 in control embryos. Note that the genes show similar spatio-temporal expression profiles within the PA CNCC and otic vesicle. (G-J) Expression of the Dlx5-lacZ reporter recapitulates the expression profiles of Dlx5 and Dlx6 in E9.5, E10.5, E11.5 embryos, but showing better signal in the posterior PAs (PA3–6). In clarified E14.5 Dlx5lacZ/+ foetuses, β-gal expression is activated in the developing skeleton. Magnifications of the ear in (J’) and of the laryngeal regions in (J”) (n = 3 each condition). Abbreviations: e, eye; hy, hyoid cartilage; lb, limb; lr, laryngeal cartilages; mb, mandible; mx, maxilla; ov, otic vesicle; PA, pharyngeal arch; sc, semicircular canals; vt, vertebrae. Scale bar in F for A, D, G 150 µm, for B, E, H 200 µm, for C, F, I 300 µm, for J 500 µm, for J’-J” 150 µm. (TIF) [file pone.0337426.s001.tif]

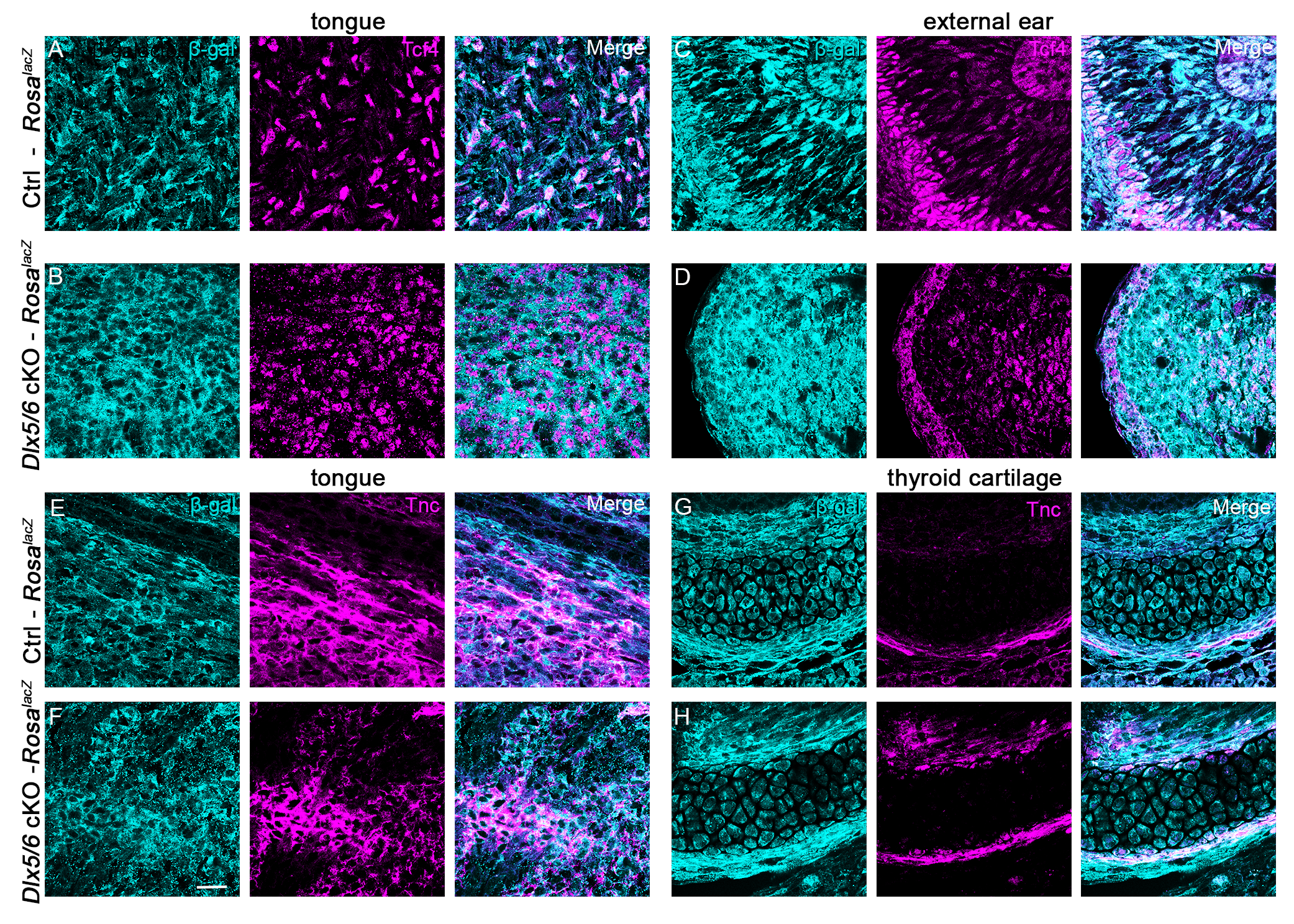

Supplement: S2 Fig — (A-H) Immunofluorescence stainings for β-gal and for Tcf4 and Tnc, markers of CNCC-derived fibroblasts and tendons at the tongue, external ear and thyroid levels in control and Dlx5/6 mutant conditions. (A-B, E-F). In the tongue, β-gal-positive cells show defects of patterning but keep their fibroblast and tendinous identities (C-D), as they do at external ear and thyroid levels (C-D, E-H) (n = 3 each condition, control genotypes: Dlx5/6flox/+; RosalacZ/+). Scale bar in F for A-H 20 µm. (TIF) [file pone.0337426.s002.tif]

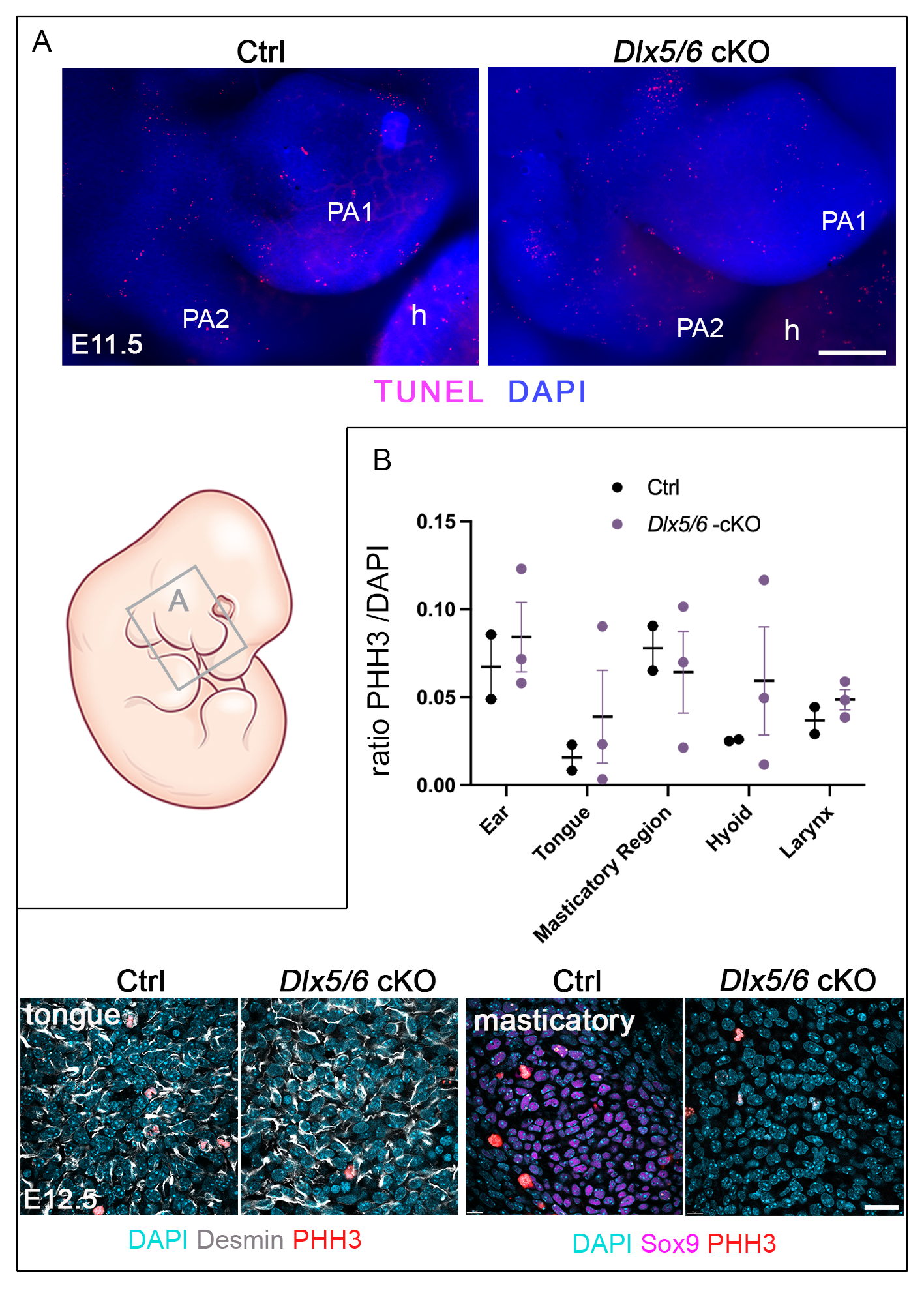

Supplement: S3 Fig — (A) TUNEL assay on whole-mount control and mutant embryos at E11.5. A Similar number of apoptotic cells were identified within the maxillary and mandibular prominences in control and mutant specimens (n = 3 each condition, control genotype: Dlx5/6flox/flox). Abbreviations: h, heart; PA1–2, pharyngeal arches 1–2. Scale bar in A 200 µm. (B) Quantitative analysis indicates no significant difference using the non-parametric Mann–Whitney test in the proportion of PHH3-positive proliferative cells between E12.5 control (n = 2, control genotype: Sox10cre/+; Dlx5/6flox/+) and mutant (n = 3) specimens across the regions of interest shown in panels as examples. Scale bar in B 20 µm. Raw data quantifications are presented in (S4 Table). (TIF) [file pone.0337426.s003.tif]

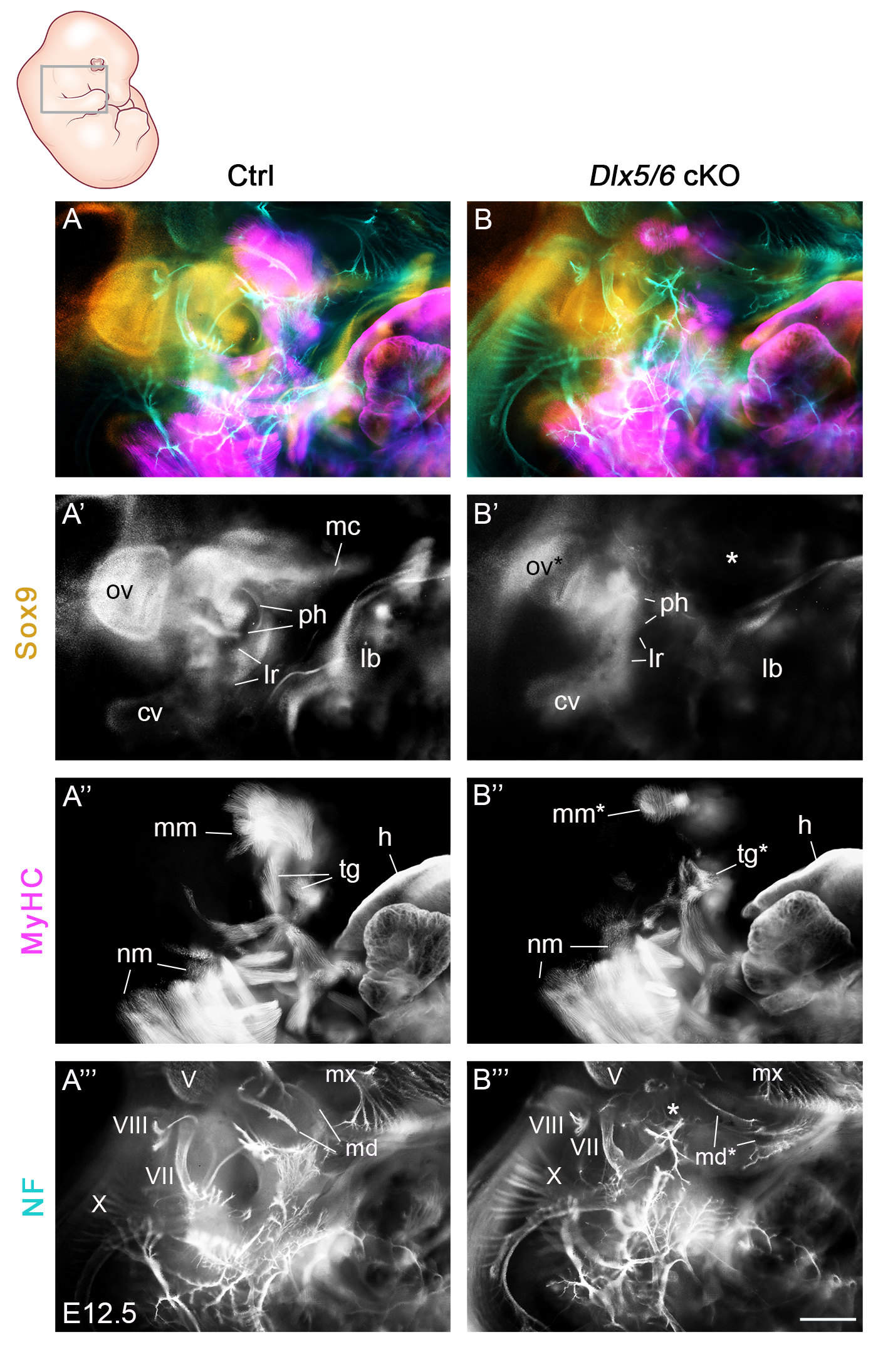

Supplement: S4 Fig — (A-B) In toto immunofluorescence staining of control and mutant embryos at the E12.5 for Sox9, MyHC and NF that label the developing cartilaginous, muscular and nervous systems respectively, at the level indicated on the scheme at the top. (A’-B’) In mutants, the chondrogenic condensations of the otic vesicle, the pharynx and the larynx appear already defective while the Meckel cartilage is undetectable compared to control (B’, white asterisk). (A”-B”) The mutant also presents a severe reduction of the masticatory muscle precursor and tongue defects, while the neck and heart musculature appear normal. (A”’-B”’). At late embryonic stage, the vestibulocochlear nerve (VIII) is slightly reduced, and the transformed mandibular branch of the trigeminal nerve shows aberrant distal arborization associated with a defect of neuronal projection towards the masticatory muscle precursor (B,” white asterisk). All structures presenting a mutant phenotype are noted with an asterisk (n = 2 each condition, control genotype: Dlx5/6flox/+). Abbreviations: cv, cervical vertebrae; h, heart; lb, limb; lr, larynx region; mc, Meckel cartilage precursor; md, mandibular branch of the trigeminal ganglion; mm, masticatory muscle precursor; mx, maxillary branch of the trigeminal ganglion; nm, neck muscles; ov, otic vesicle; ph, pharynx region; tg, tongue precursor. Scale bar in B”’ for A-B”’ 400 µm. (TIF) [file pone.0337426.s004.tif]
