# Supplementary material for: Pleiotropic function of Dlx5/6 in the development of mammalian vocal and auditory organs
Source: PLoS One. 2025 Dec 2;20(12):e0337426. doi: 10.1371/journal.pone.0337426 (PMC12671821; doi:10.1371/journal.pone.0337426)
Supplement: S1 Table — (PDF) [file pone.0337426.s005.pdf]

S1 Table. List of primary and secondary antibodies used in the study

Primary antibody	Isotype	Dilution	Source
Anti-β-galactosidase	chick IgG	1:1000	Abcam (ab9361)
Anti-Sox9	rabbit IgG	1:1000	Merck (ab5535)
Anti-Myosin Heavy Chain	mouse IgG2a	1:20	DHSB (A4.1025)
Anti-Tenascin	rat IgG1	1:200	Life Tech (MA126778)
Anti-Tcf4	rabbit IgG	1:100	Ozyme (C48H11)
Anti-Desmin	rabbit IgG	1:100	Abcam (Y66 abcam ab32362)
Anti-NF	mouse IgG1	1:20	DHSB (2H3)
Anti-PHH3	mouse IgG1	1:300	Milipore (ab05 806)

Secondary antibody	Dilution	Fluorochrome	Source
Goat anti-IgG1 mouse	1:500	AffiniPure Cy3	Jackson Immunoresearch (115-167-185)
Goat anti-IgG2a mouse	1:500	AffiniPure 647	Jackson Immunoresearch (115-605-206)
Goat anti-IgG chick	1:500	Alexa Fluor 488	Life Tech (A11039)
Goat anti-IgG rabbit	1:500	Alexa Fluor 488<br>Alexa Fluor 555<br>Alexa Fluor 647	Life Tech (A11070)<br>Life Tech (A21430)<br>Life Tech (A21245)
Goat anti-IgG rat	1:500	Alexa Fluor 555	Life Tech (A21434)
