# Supplementary material for: Pleiotropic function of Dlx5/6 in the development of mammalian vocal and auditory organs
Source: PLoS One. 2025 Dec 2;20(12):e0337426. doi: 10.1371/journal.pone.0337426 (PMC12671821; doi:10.1371/journal.pone.0337426)
Supplement: S2 Table — (PDF) [file pone.0337426.s006.pdf]

S2 Table. Microscopes and acquisition parameters

System	Type	Objective (NA)	Lasers
LSM 980 – AiryScan 2 detector Zeiss	Confocal - AiryScan	EC-Plan Neofluar 10X dry (0.3 NA) or Plan-Apochromat water 20X (0.8 NA) or 63X C-Apochromat oil (1.2 NA)	405 – 488 – 561 – 639 nm
Macro-Apotome AxioZoom V16 Zeiss	Macroscope	PlanNeoFluar Z 1.0X oil (0.25 NA) or PlanNeoFluar Z 2,3X (0.57 NA)	Excitation by fluorescent lamp with adapted filter block (DAPI, GFP, DsRed, Cy5)
Lightsheet Alpha3 PhaseView	Lightsheet	XLPLN 10X Olympus objective (0.6 NA) multi-immersion refractive index adaptive collar (RI 1.33 – 1.52)	405 – 488 – 561 – 635 nm
