# Supplementary material for: Pleiotropic function of Dlx5/6 in the development of mammalian vocal and auditory organs
Source: PLoS One. 2025 Dec 2;20(12):e0337426. doi: 10.1371/journal.pone.0337426 (PMC12671821; doi:10.1371/journal.pone.0337426)
Supplement: S3 Table — (PDF) [file pone.0337426.s007.pdf]

S3 Table. Composition of solutions used for *in situ* hybridization

Hyb Mix (Hb)

Formamide	50%
SSC 20X	5X
Yeast RNA (20 mg/ml)	0,1 mg/ml
Heparin (20 mg/ml)	0,1 mg/ml
CHAPS 5%	0,5%
EDTA 0,5M	5mM
Tween 10%	0,1%
H2O mili Q	

Wash Buffer (WB)

Formamide	50%
SSC 20X	5X
CHAPS 5%	0,5%
EDTA 0,5M	5mM
Tween 10%	0,1%
H2O mili Q	

Blocking Buffer (BB)

Levamisol 200X	1X
NGS 100%	10%
TBST	

Antibody Solution (SAc)

Levamisol 200X	1X
NGS 100%	1%
Antibody anti-DIG	1/2000
TBST	

NTMT

NaCl 5 M	100mM
Tris pH 9,5 1M	100mM
MgCl2 1 M	50mM
Tween 10%O	0,1%
H2O mili Q	

TBST 20 X

NaCl	1.4 M
KCl	27 mM
Tris-HCL 1M	0,25 M
Tween-20 (100%)	1%
H2O mili Q	
