# Supplementary material for: Pleiotropic function of Dlx5/6 in the development of mammalian vocal and auditory organs
Source: PLoS One. 2025 Dec 2;20(12):e0337426. doi: 10.1371/journal.pone.0337426 (PMC12671821; doi:10.1371/journal.pone.0337426)
Supplement: S4 Table — (PDF) [file pone.0337426.s008.pdf]

S4 Table. Raw data of PHH3 and DAPI cells quantification shown in S3 Fig.

	**Ctrl#1 (*Sox10*$^{Cre/+}$ *; Dlx5/6*$^{f/+}$)**								
	PHH3				DAPI				**PHH3/DAPI Ratio**
	Section 1	Section 2	Section 3	**Total**	Section 1	Section 2	Section 3	**Total**	
Ear	18	21	20	59	238	214	237	689	0.08563135
Tongue	5	7	3	15	195	224	235	654	0.02293578
Masticatory Region	25	37	24	86	322	344	283	949	0.090621707
Hyoid	6	12	6	24	322	301	298	921	0.026058632
Larynx	13	16	5	34	197	275	293	765	0.04444444

	**Ctrl#2 (*Sox10*$^{Cre/+}$ *; Dlx5/6*$^{f/+}$)**								
	PHH3				DAPI				**PHH/DAPI Ratio**
	Section 1	Section 2	Section 3	**Total**	Section 1	Section 2	Section 3	**Total**	
Ear	10	9	14	33	197	278	199	674	0.048961424
Tongue	0	3	2	5	195	201	202	598	0.008361204
Masticatory Region	22	16	14	52	229	298	270	797	0.065244668
Hyoid	4	8	8	20	274	234	291	799	0.025031289
Larynx	5	4	8	17	196	202	186	584	0.02910959

	***Dlx5/6* -cKO#1 (*Sox10*$^{Cre/+}$ *; Dlx5/6*$^{f/f}$)**								
	PHH3				DAPI				**PHH3/DAPI Ratio**
	Section 1	Section 2	Section 3	**Total**	Section 1	Section 2	Section 3	**Total**	
Ear	14	16	14	44	187	207	220	614	0.071661238
Tongue	0	0	2	2	205	193	217	615	0.003252033
Masticatory Region	6	5	1	12	212	174	177	563	0.021314387
Hyoid	1	4	2	7	214	193	194	601	0.011647255
Larynx	8	14	7	29	220	173	206	599	0.04841402

	Dlx5/6 -cKO#2 (*Sox10*$^{Cre/+}$ *; Dlx5/6*$^{f/f}$)								
	PHH3				DAPI				PHH3/DAPI Ratio
	Section 1	Section 2	Section 3	**Total**	Section 1	Section 2	Section 3	**Total**	
Ear	32	35	20	87	250	226	231	707	0.123055163
Tongue	23	14	21	58	224	205	213	642	0.090342679
Masticatory Region	17	17	24	58	285	274	271	830	0.069879518
Hyoid	8	9	13	30	200	187	218	605	0.049586777
Larynx	7	19	20	46	234	265	282	781	0.05889885

	Dlx5/6 -cKO #3 (*Sox10*$^{Cre/+}$ *; Dlx5/6*$^{f/f}$)								
	PHH3				DAPI				PHH3/DAPI Ratio
	Section 1	Section 2	Section 3	**Total**	Section 1	Section 2	Section 3	**Total**	
Ear	18	12	15	45	266	250	259	775	0.058064516
Tongue	4	3	7	14	218	211	175	604	0.023178808
Masticatory Region	25	10	34	69	235	228	216	679	0.101620029
Hyoid	21	29	26	76	221	227	203	651	0.116743472
Larynx	7	10	11	28	192	282	252	726	0.03856749
