# Supplementary material for: Pleiotropic function of Dlx5/6 in the development of mammalian vocal and auditory organs
Source: PLoS One. 2025 Dec 2;20(12):e0337426. doi: 10.1371/journal.pone.0337426 (PMC12671821; doi:10.1371/journal.pone.0337426)
Supplement: S1 Appendix — Interactive 3D PDF of the control phenotype reconstructions of vocal tract and auditory systems to complete data shown in Fig 1E–H’. (PDF) [file pone.0337426.s009.pdf]

Interactive 3D reconstruction of a control foetus (E17.5)

All structures

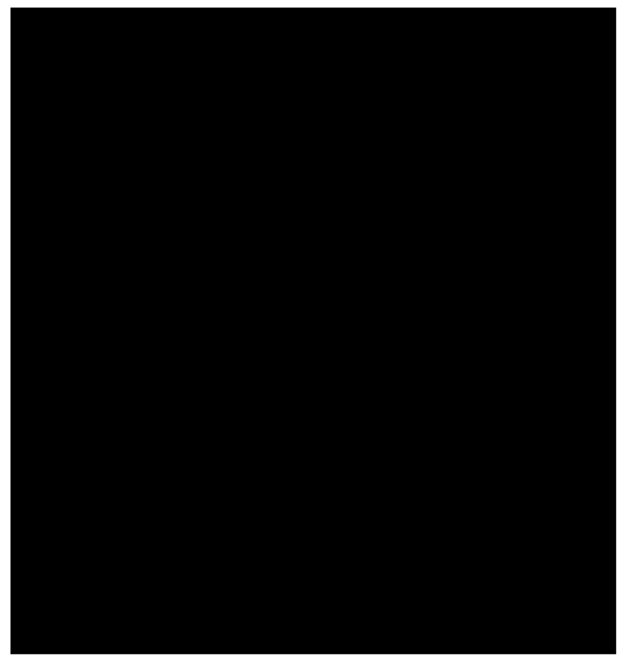

Preset views

ventral

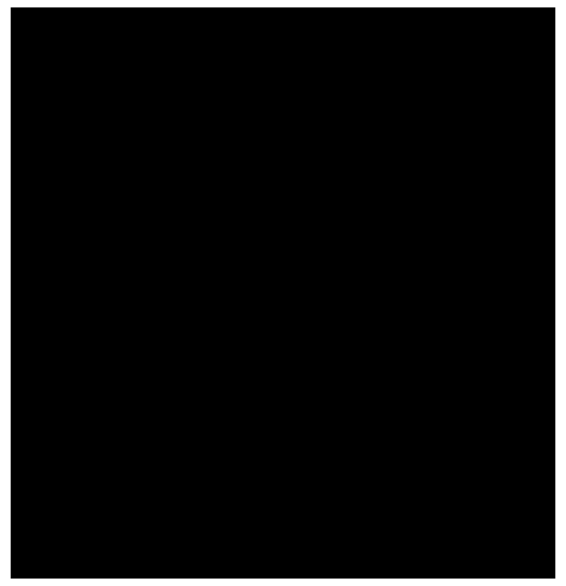

dorsal

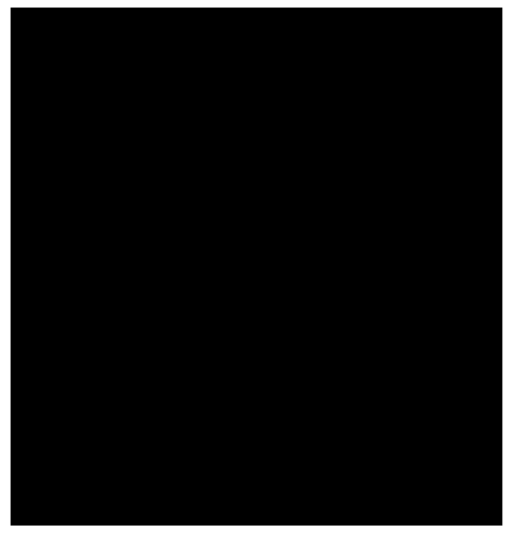

lateral

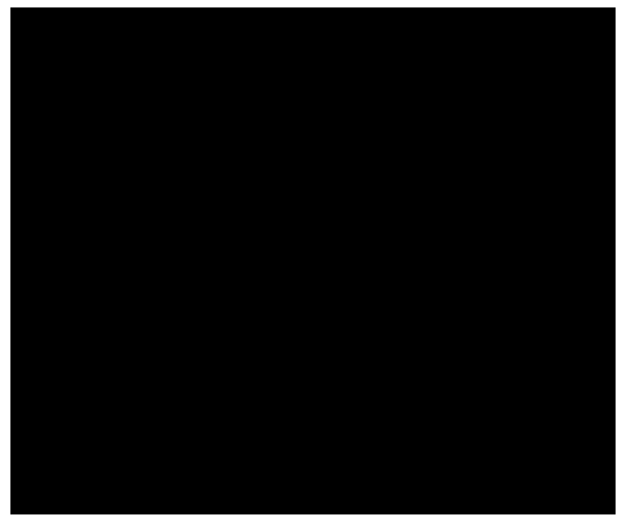

medial

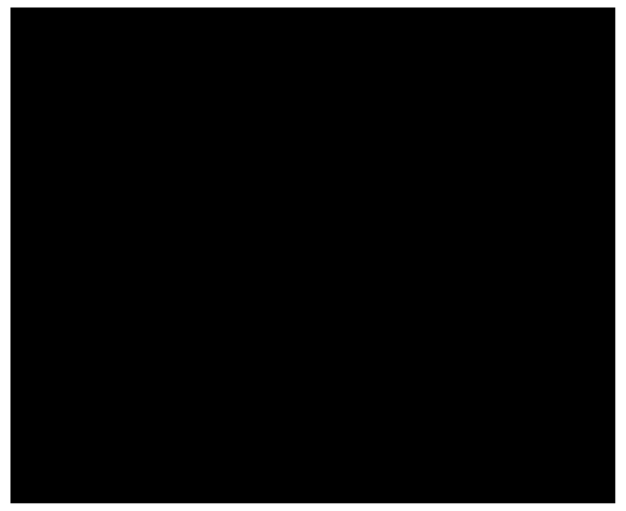

Preset structures

laryngeal and hyoid cartilages

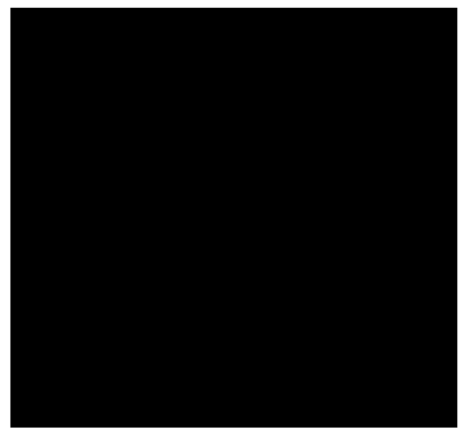

laryngeal and pharyngeal muscles

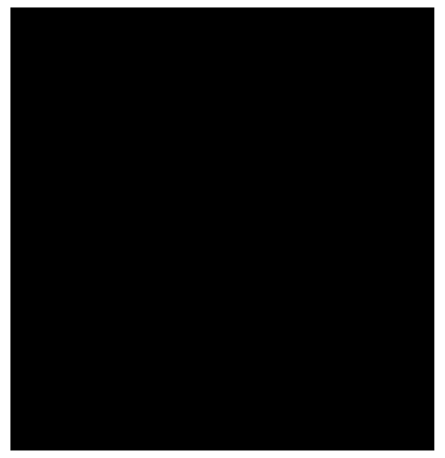

jaws + teeth + palate

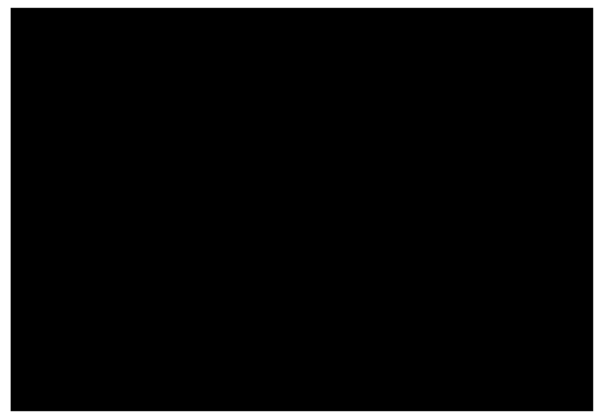

all muscles

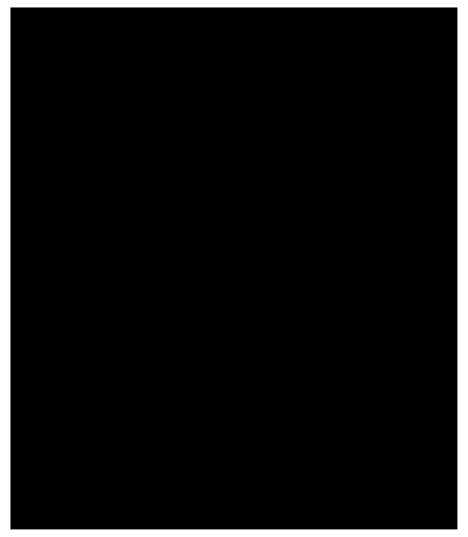

middle ear and Meckel cartilage

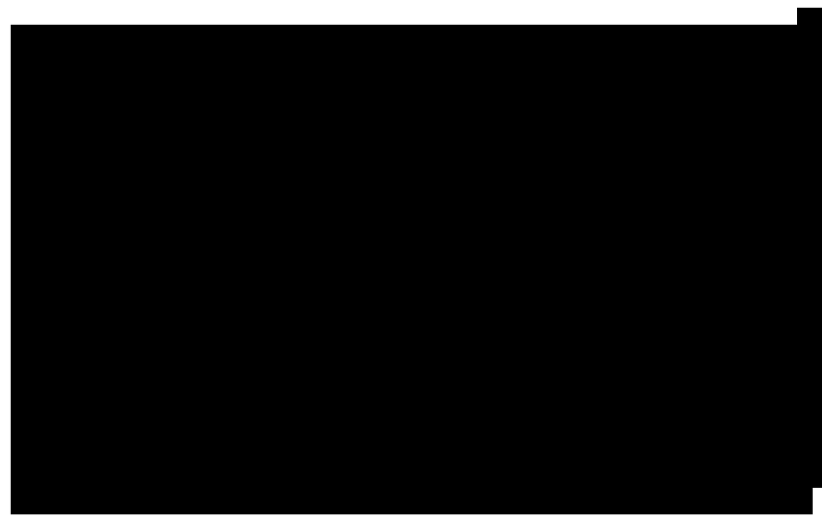

inner ear

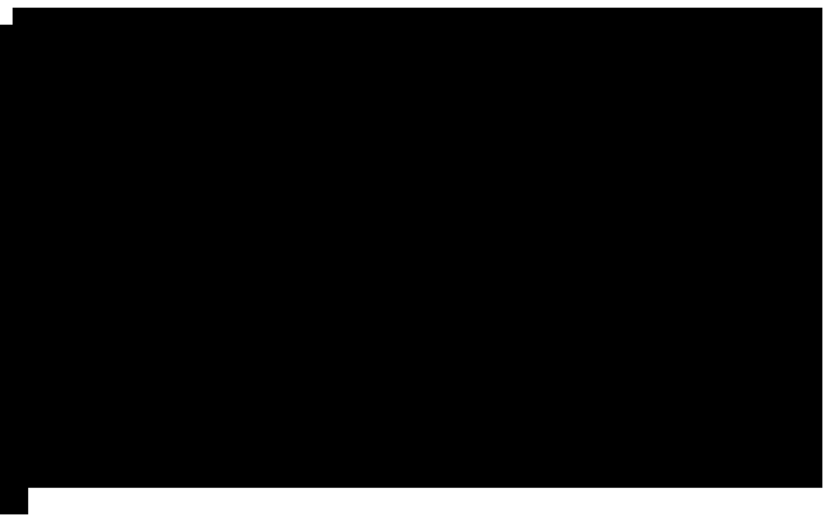

Legend box: Cartilages Jaws Muscles
