# Supplementary material for: Pleiotropic function of Dlx5/6 in the development of mammalian vocal and auditory organs
Source: PLoS One. 2025 Dec 2;20(12):e0337426. doi: 10.1371/journal.pone.0337426 (PMC12671821; doi:10.1371/journal.pone.0337426)

# Interactive 3D reconstruction of a Sox10-inactivated foetus (E17.5)

## All structures

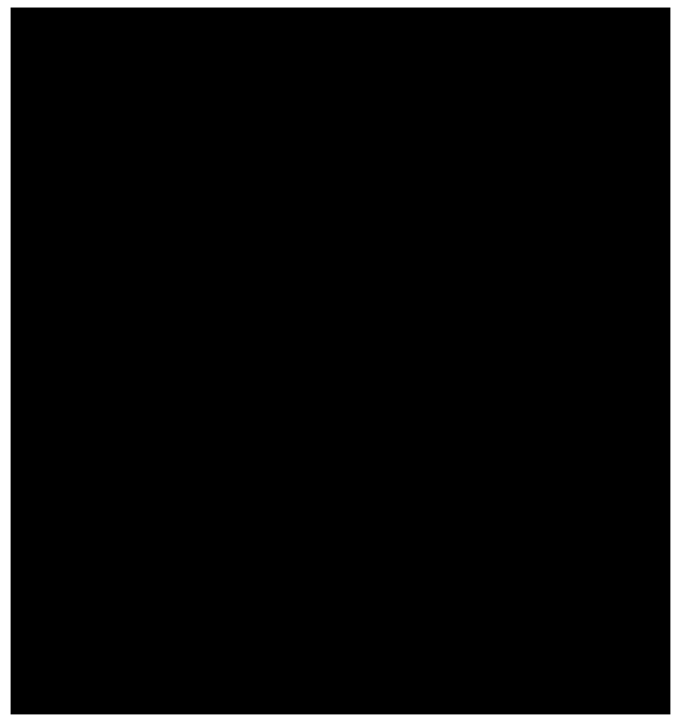

Legend box:

Cartilages

Bones

Muscles

## Preset views

ventral

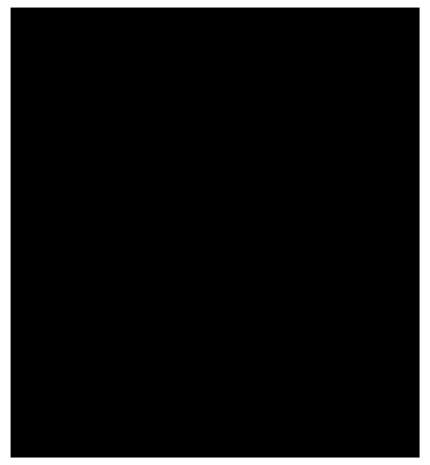

dorsal

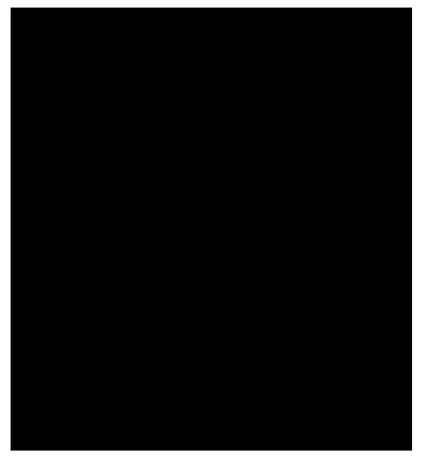

lateral

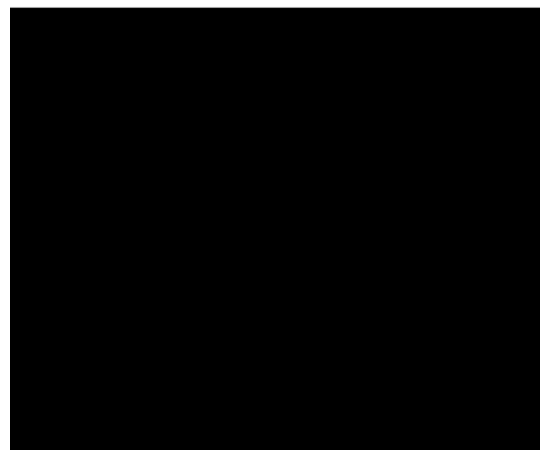

medial

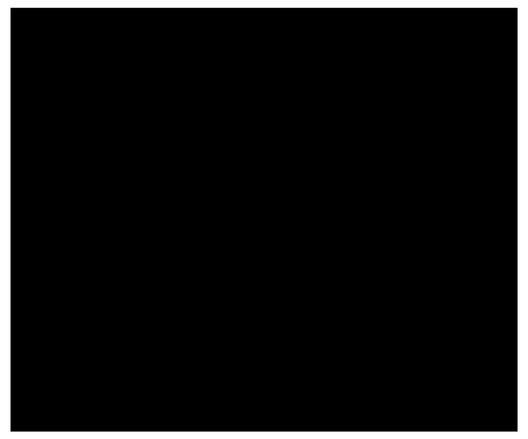

## Preset structures

hyoid + laryngeal cartilages

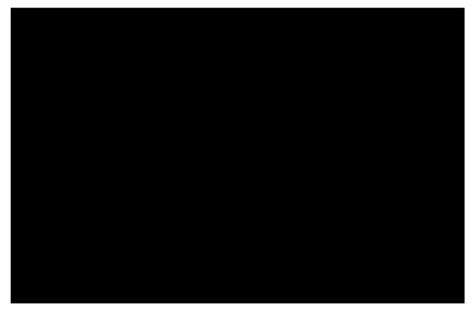

pharyngeal + laryngeal muscles

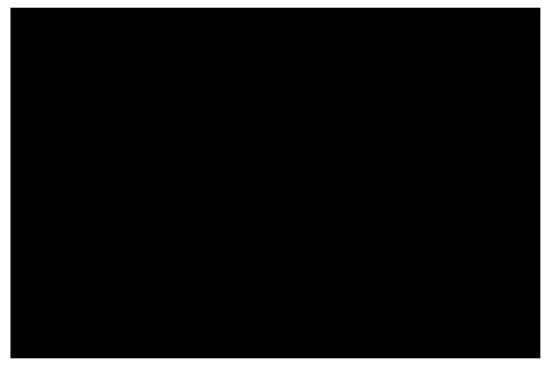

jaws + teeth + palate

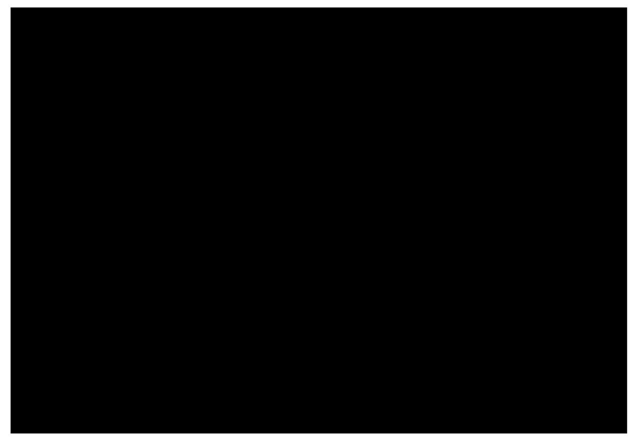

all muscles

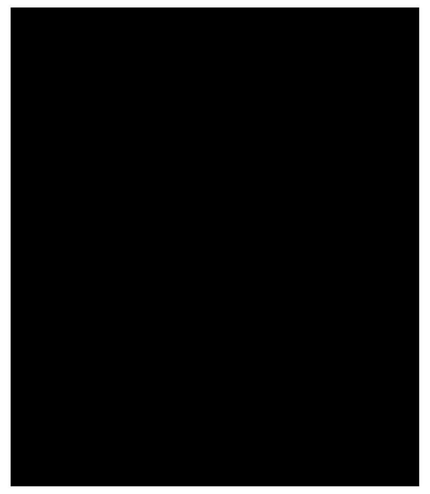

Otic capsule + middle ear

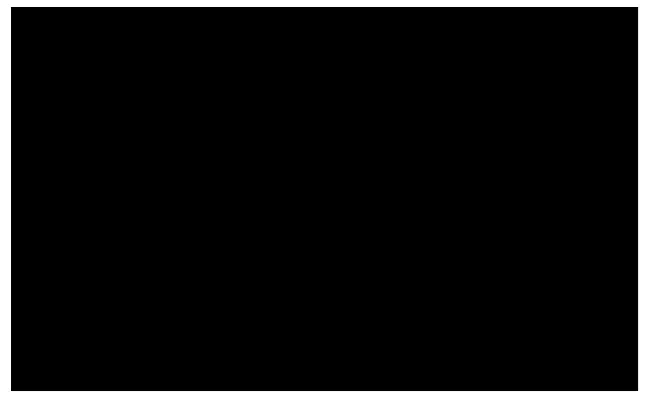

inner ear

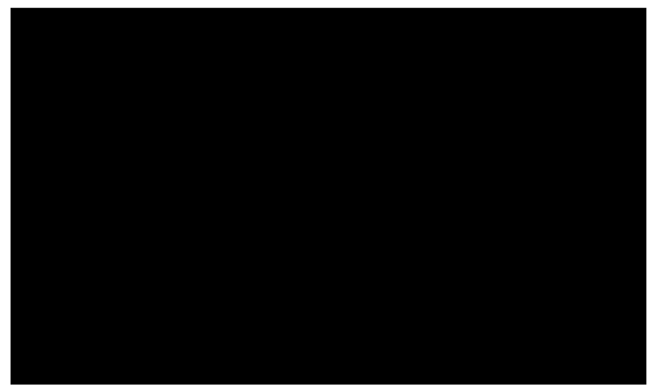

Supplement: S2 Appendix — (PDF) [file pone.0337426.s010.pdf]
